# Short-term responses of unicellular planktonic eukaryotes to increases in temperature and UVB radiation

**DOI:** 10.1186/1471-2180-12-202

**Published:** 2012-09-11

**Authors:** Isabelle Domaizon, Cécile Lepère, Didier Debroas, Marc Bouvy, Jean Francois Ghiglione, Stephan Jacquet, Yvan Bettarel, Corinne Bouvier, Jean Pascal Torréton, Francesca Vidussi, Behzad Mostajir, Amy Kirkham, Emilie LeFloc’h, Eric Fouilland, Helene Montanié, Thierry Bouvier

**Affiliations:** 1 INRA, UMR 42 CARRTEL, 75 avenue de Corzent, BP511, Thonon-les-bains, F-74200, France; 2 Clermont Université, Université Blaise Pascal, CNRS, UMR 6023, LMGE, BP 10448, Avenue des Landais, Clermont Ferrand, F-63000, France; 3 Université Montpellier 2, UMR 5119 ECOSYM, CNRS, IRD, Ifremer, Université Montpellier 1, Place E Bataillon, cc 093, Montpellier cedex 5, 34095, France; 4 CNRS, UMR 7621, LOMIC, Observatoire Océanologique, Avenue de Fontaulé, Banyuls/mer, F-66651, France; 5 LIttoral ENvironnement et Sociétés (LIENSs) - UMR 6250, Université de La Rochelle, Institut du Littoral et de l’Environnement (ILE), 2 rue Olympe de Gouges, La Rochelle, 17 000, France

**Keywords:** Small eukaryotes, Molecular diversity, Temperature, UVB radiation, Microcosms experiment, Mediterranean lagoon

## Abstract

**Background:**

Small size eukaryotes play a fundamental role in the functioning of coastal ecosystems, however, the way in which these micro-organisms respond to combined effects of water temperature, UVB radiations (UVBR) and nutrient availability is still poorly investigated.

**Results:**

We coupled molecular tools (18S rRNA gene sequencing and fingerprinting) with microscope-based identification and counting to experimentally investigate the short-term responses of small eukaryotes (<6 μm; from a coastal Mediterranean lagoon) to a warming treatment (+3°C) and UVB radiation increases (+20%) at two different nutrient levels. Interestingly, the increase in temperature resulted in higher pigmented eukaryotes abundances and in community structure changes clearly illustrated by molecular analyses. For most of the phylogenetic groups, some rearrangements occurred at the OTUs level even when their relative proportion (microscope counting) did not change significantly. Temperature explained almost 20% of the total variance of the small eukaryote community structure (while UVB explained only 8.4%). However, complex cumulative effects were detected. Some antagonistic or non additive effects were detected between temperature and nutrients, especially for Dinophyceae and Cryptophyceae.

**Conclusions:**

This multifactorial experiment highlights the potential impacts, over short time scales, of changing environmental factors on the structure of various functional groups like small primary producers, parasites and saprotrophs which, in response, can modify energy flow in the planktonic food webs.

## Background

Small-sized plankton plays critical roles in aquatic systems, mostly as major contributors to production and biomass, and as key players driving carbon and nutrient cycles [1,2]. The study of the gene coding for 18S rRNA has brought opportunities to investigate the eukaryotic composition in the smallest size fraction in various aquatic systems, independently of morphological identification and cultivation [3-7]. The molecular characterization of small (pico and/or nano) eukaryotic assemblages has highlighted an unexpected phylogenetic and functional diversity (e.g. [8-11]), and many important questions are now emerging about the *in situ* dynamics of diverse eukaryotic groups, and the regulatory factors that drive changes in their structure.

A few studies have investigated the effects of structuring factors on the molecular diversity of small eukaryotes, and shown that trophic status, predation by met zooplankton, and/or viral lytic activity are involved in the regulation of the eukaryotic microbial assemblage [5,12-15]. However, combined effects of physical factors, such as water temperature and UVB radiation (UVBR: 280–320 nm) are still poorly investigated. It is recognized that either temperature or UVBR increases can modify microbial dynamics and structure at various levels (species, population, trophic network) (e.g. [16-20]). Nevertheless, previous investigations have generally focused on only one specific stressor and little is known about the combined effects of climatic and anthropogenic stressors on diversity and food web structure. Since these stressors are expected to exert complex interactive effects [21-23], multi-factorial studies are required to improve the understanding of the mechanistic basis underlying ecological responses of planktonic food webs to these regulatory factors. A series of enclosure experiments using natural microbial communities from the Mediterranean Thau lagoon were recently performed to assess the response of microbial communities to top-down and bottom-up control under various simulated climatic conditions (temperature and UVBR) [24]. This study showed a much larger effect of temperature than UVBR on bacterial dynamics. In addition to this study, in order to describe the composition of small eukaryotes and potentially to observe changes in their structure, we used a similar microcosm experiment to tease apart the effects of single and combined increase of temperature (+3°C) and UVBR (+20%), at two different nutrients levels. Here, we investigate short-term responses of both pigmented and non-pigmented small eukaryotes (size fraction <6 μm) to these simulated climatic conditions by using morphological and molecular methods (18S rRNA gene sequencing and a fingerprint technique: Capillary Electrophoresis Single Strand Conformation Polymorphism CE-SSCP).

The increases in temperature and UVBR tested in this study correspond to the mean temperature increase expected in the Mediterranean region by 2080–2099 (IPCC 2007) and the high-UVBR scenario for the European region during spring in future years [22]. This approach enables us to describe the short term responses of eukaryotic community assemblages when exposed to these drivers during the productive spring season. The changes induced by these regulatory factors could be detected at different taxonomic levels thanks to the coupling of morphological and molecular approaches.

## Methods

### Experimental design

The four-day experiment (20–23 April 2006) was conducted on the Mediterranean platform for Marine Ecosystems Experimental Research (MEDIMEER) located in Sète (France) on the shore of the Thau lagoon (43°24’49”N, 3°41’19”E). The experimental platform was composed of submerged enclosures (1.2 m diameter and 2 m depth) which allowed the isolation of up to 2,000 L and the simulation of UVBR and temperature increases in order to study the responses of pelagic communities to these manipulated factors simultaneously. The regulations of UVBR and temperature are performed with high frequency monitoring following the *in situ* temperature and natural incident UVBR (see details in supplementary data; full description in Nouguier *et al*. [25]).

**Figure 1:**
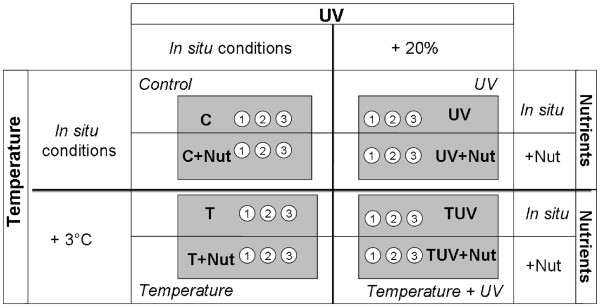
Crossed factorial experimental design conducted to assess the effects of the three regulatory factors: (Temperature, UVB radiation and nutrient increases).

Four enclosures, filled with lagoon surface-water at random, were used as incubators for the 2 L experimental bags (UV-permeable sterile Whirl Pack® polyethylene bags incubated at subsurface) in which microbial communities were isolated. The factorial experimental design constituted eight different treatments (each being tested in three replicates): **C**: control, **C + Nut**: control with nutrient addition, **UV**: UVBR increase (+20%), **UV + Nut**: UVBR increase (+20%) and nutrient addition, **T**: temperature increase (+3°C), **T + Nut**: temperature increase (+3°C) and nutrient addition, **TUV**: temperature (+3°C) and UVBR (+20%) increases, **TUV + Nut**: temperature (+3°C) and UVBR increases (+20%) and nutrient addition (Figure 1).

In order to fill the 24 Whirl Pack bags, 100 L subsurface lagoon water was pumped and pre-filtered through 6-μm-pore-size polycarbonate membranes (47 mm in diameter) in order to isolate the smallest planktonic fraction. This water sample (<6 μm) was equally distributed into 24 sterile Whirl Pack® polyethylene bags. 12 of these experimental bags received nutrients addition at time zero, while the others were kept without nutrient addition. The set bags which represented the enriched nutrient conditions were obtained by addition of a mixture of leucine (C and N) and phosphate in order to maintain a substrate C:N:P molar ratio close to that of marine bacteria [26] as described in Bouvy *et al.*[24]. The bags with and without nutrient addition exhibited concentrations of 0.20 μM and 0.07 μM of PO_4,_ respectively. The two levels of P concentration mimicked natural fluctuations in coastal lagoon waters. These concentrations were chosen to be relevant to phosphorus concentrations recently measured in Thau lagoon (a general decrease over the past 30 years has led to low values of soluble reactive phosphorus: i.e. from 3 μM to undetectable values (<0.03 μM in winter) [27]).

Since nutrients usually refer to inorganic nutrients, it should be noted that in this study, “nutrients” actually refer to “nutrients and organic source of C and N”.

The incubation time (4 days) and experimental volume (2 L) were chosen to be consistent with the generation time of microorganisms, as validated in other experimental studies [28-31]. In the present study, the composition of unicellular eukaryotes was studied at T0 and T96h. The data provided by Bouvy *et al.*[24] regarding the evolution of abundances of the main biological communities (i.e. bacteria, viruses, heterotrophic flagellates) at 3 sampling times (T0, T48h, T96h) under the same experimental conditions as ours, informed this choice.

### Measurement of abiotic parameters

Temperature was continuously measured using thermistor probes (Campbell Scientific 107). Incident UVBR (280–320 nm) was constantly monitored by a UVB radiometer (SKU 430, Skye instruments). During the experiment, temperature varied between 15.7°C and 17.2°C (and between 18.7°C and 20.2°C in ‘+3°C’ treatments), while incident UVB radiations (280–320 nm), which were measured around local zenith time, varied between 150 and 185 μWcm^-2^ (Table 1). At T0 and T96 h, samples were taken for abiotic analysis. A volume of 80 ml of water was filtered on pre-combusted glass fiber filters (GF/F, Whatman) and stored at −20°C until nitrate and phosphate concentrations were measured, following standard nutrient analysis methods [32].

**Table 1:** **Environmental conditions (temperature, salinity, chlorophyll*a*concentration, natural UVBR intensities) during the four days experiment**

***Environmental conditions during the 4 days of study***	
Period	Spring (18–24 April)
*In situ* Temperature	15.7°C to 17.2°C
*In situ* Salinity	Approx. 36
*In situ* Chl a	Approx. 1 μg/L
*In situ* maximum UVBR incidentsN (local zenith time)	150 to 185 μW/cm2

### Bacterial and viral counting by flow cytometry

At T0 and T96h, 5 ml of water was collected from each of the polyethylene bags for flow cytometry counts. Picocyanobacteria, heterotrophic bacteria and viruses were counted using a FACSCalibur flow cytometry (Becton Dickinson) equipped with an air-cooled laser providing 15 mW at 488 nm. For photosynthetic-cells (*i.e.* picocyanobacteria) neither fixative nor fluorochrome were used. Samples were stored at <4°C until analysis, which was performed within 2 h of sampling in field laboratories. Analysis was therefore performed on fresh samples, to which a suspension of 1-μm beads (Molecular probes) was added, generally for 4 to 8 minutes in order to obtain >20,000 events. For the analysis of bacteria and viruses, 1 mL fixed (glutaraldehyde 0.5% final concentration) sub-samples were incubated with SYBR Green I (Molecular Probes, Eugene, OR, USA) at a final concentration of 1/10,000 for 15 min at room temperature in the dark. The cytometry flow counts were performed as described in Brussard *et al*. [29].

### Small eukaryotes microscopy observation

For enumeration of non-pigmented and pigmented eukaryotes, water samples (100 mL) taken at T0 and T96h were fixed with glutaraldehyde (1% final concentration) and stored at 4°C for 24 h. 20 to 25 ml of each preserved water sample was stained with DAPI (final concentration, 15 μg mL^−1^) for 15 min, filtered onto a black Nuclepore filter (0.8 μm-pore-size), stored at −20°C, and counted under an epifluorescence microscope with UV excitation (modified from Boenigk *et al.*[33]). Under UV light (350/461 nm), the eukaryotic cell nucleus appears as a separate organelle, while prokaryotic organisms appear as cells uniformly stained without visible nuclei. The blue and green light excitations were used to reveal pigmented cells.

### Molecular analysis of small eukaryotes

#### Sampling and preservation

Water samples from each treatment were taken at the beginning and at the end of the experiment. The microbial biomass was collected on 0.2 μm pore size polycarbonate membranes (Millipore) under very low vacuum (<20 mbar) to prevent cell damage. Filters were then stored at −80°C until nucleic acid extraction.

#### Nucleic acid extraction

Nucleic acid extraction was performed as described by Lefranc *et al*. [34] and extracts were stored at −20°C until analysis.

#### Capillary electrophoresis – single strand conformation polymorphism (CE-SSCP)

Nucleic acids from each sample were used as templates for PCR amplification of the 18S rRNA gene with primers Uni1392r (5’-ACG-GGC-GGT-GTG-TRC-3’) labelled at the 5’-end with phosphoramidite [35] and Euk1209f (5’-CAG-GTC-TGT-GAT-GCC-CGC-3’) [36]. Each 25 μL reaction mixture contained 50 μM of each primer, 1X Pfu reaction buffer, 20 mM dNTPs, 1.0 U of Pfu DNA polymerase (Promega) and 0.1 μg of template DNA. PCR amplification was performed with a Rob cycler (Stratagene) under the following conditions: an initial denaturation step of 94°C for 2 min, followed by 10 touchdown cycles of denaturation at 94°C for 1 min, annealing at 65°C (with the temperature decreasing 1°C each cycle) for 1 min, and extension at 72°C for 1 min, followed by 15 cycles of 94°C for 1 min, 55°C for 1 min and 72°C for 1 min, and a final elongation step at 72°C for 10 min. The TET-labelled PCR products were quantified by visualization in ethidium bromide-stained agarose gels (2%) and diluted in sterile TE (10 mM Tris, 1 mM EDTA) in order to obtain around 10 ng mL^–1^ of PCR product. One μL of the dilution was mixed with 18.9 μL of formamide (Applera Corp. Norwalk, Connecticut) and 0.1 μL of the internal size standard Gene-Scan-400 Rox (Applied Biosystems), denatured at 94°C for 5 minutes, and immediately cooled on ice for 10 minutes before electrokinetic injection (5 s, 12 kV) into a capillary tube (47 cm x 50 μm) filled with 5.6% of Gene Scan polymer in a ABI Prism 310 Genetic analyser (Applied Biosystems). Electrophoresis was carried out and data were collected as described in Sauret *et al*. [37].

#### Eukaryotic rRNA genetic libraries

Environmental DNA extracts were also used to construct the 18S rRNA gene clone libraries. The eukaryote-specific primers Ek-1 F (5’-CTG-GTT-GAT-CCT-GCC-AG-3’) and Ek-1520R (5-CYG-CAG-GTT-CAC-CTA-C-3’) were used for PCR amplification [38]. The PCR mixture (50 μL) contained about 10 ng of environmental DNA, 200 μM of each deoxynucleoside triphosphate, 2 mM MgCl_2_, 10 pmol of each primer, 1.5 U of *Taq* DNA polymerase (Eurobio) and the PCR buffer supplied with the enzyme. Reactions were carried out in an automated thermocycler (MJ Research PTC 200-cycler) with the following cycle: initial denaturation at 95°C for 5 min, 30 cycles of denaturation at 95°C for 1 min, annealing at 57°C for 1 min, and extension at 72°C for 1 min 30 s, and a final extension at 72°C for 10 min. PCR products (at least four 50 μL samples) from the triplicate samples of each experimental condition were pooled, precipitated with ethanol–sodium acetate and re-suspended in 50 μL of sterile water. Clone libraries were constructed for the T0 control and for each of the eight treatments at T96 h using a TOPO TA cloning kit (Invitrogen, Carlsbad, CA) with PCR vector 2.1 according to the manufacturer’s instructions.

#### Phylogenetic analysis

DOTUR was used to determine operational taxonomic units (OTUs) from 18S sequences data [39] with a cut-off of 97% sequence similarity. To determine the phylogenetic affiliation, each sequence was first compared with sequences available in public databases using BLAST (National Center for Biotechnology Information and the Ribosomal Database Project) [40]. Secondly, the OTUs were aligned with complete sequences in an ARB database using the latter’s automatic alignment tool (http://www.arb-home.de) [41]. The resulting alignments were checked and corrected manually. Sequences were inserted into an optimised tree according to the maximum parsimony criteria without allowing any changes to the existing tree topology (ARB software). The resulting tree was pruned to retain the closest relatives, sequences representative of eukaryotic evolution and our clones (Additional file 1: Figure S1). The sequences were screened for potential chimeric structures by using Chimera check from Ribosomal Database project II and by performing fractional treeing of the 5' and 3' ends of the sequenced DNA fragments. The sequences reported in this paper have been deposited into Genbank (accession numbers: HQ393974 to HQ394162).

The relative distribution of OTUs in the library was used to calculate coverage values (Good’s coverage) [42] and the non-parametric richness estimator Chao1 [43] and ACE [44] which are the most appropriate indices for microbial clone libraries [45].

### Statistical analysis

#### Univariate analysis

We tested the homogeneity of the main biological parameters in experimental bags at the initial point (T0) of the experiment using an ANOVA test.

To test the effects of temperature, UV and nutrients on the abundance of all biological groups (bacteria, picocyanobacteria, viruses, heterotrophic flagellates and pigmented eukaryote abundances at T96 h), we used a three-way ANOVA test (with Bonferroni adjustment). Equality of the variances and normality of the residuals were tested by Bartlett and Shapiro-Wilk tests. The software Sigmastat^TM^ 3.1 was used for all analyses.

#### Multivariate analysis

Indirect multivariate analysis was used to compare CE-SSCP fingerprinting. Total fingerprinting area was normalized between the different CE-SSCP profiles using the internal size standard Gene-Scan-400 Rox using the SAFUM software [46]. Similarity matrices based on Bray-Curtis distances, dendrograms (complete linkage clustering) and ordination by non-metric multidimensional scaling (MDS) were then obtained by using the PRIMER 5 software (PRIMER-E, Ltd., UK). One-way analysis of similarity (ANOSIM, Primer-E) was performed on the same distance matrix to test the null hypothesis that there was no difference between eukaryotic communities from replicate samples of each condition.

#### Statistics applied to phylogenetic information

From the sequencing results, the beta-diversity was studied from the Unifrac distance (fraction of the total branch length in the phylogeny that is unique to each environment) of each sample. In order to compare eukaryotic communities from the 9 genetic libraries Unifrac (http://bmf2.colorado.edu/unifrac/index.psp; [47]) metrics were used to perform a principal coordinate analysis (PCA). The P-values matrix that compares each sample to each other sample was also performed from UNIFRAC metrics.

To investigate the relationships between changes in the eukaryote community structure (number of clones affiliated to each OTUs within main phylogenetic groups) and physic-chemical and biological parameters, we used direct multivariate canonical correspondence analysis (CCA) [48]. In addition to temperature values, UVB radiation, and nutrient concentrations, we considered the abundances of bacteria, picocyanobacteria, viruses, pigmented eukaryotes and heterotrophic flagellates as explanatory variables. CCA was calculated for the T96 h dataset using the Vegan package within the R software (http://cran.rproject.org/). A minimal set of explanatory variables associated with variation in eukaryote community structure was identified, allowing us to exclude the most redundant explanatory variables. Forward selection was performed to identify environmental variables that could explain a significant portion of the variation in small eukaryote structure (P < 0.05) at T96 h. Eigen values for site scores, biplot and diversity data were plotted to illustrate the associations between these data [49].

## Results

### Initial conditions

#### Biological and chemical parameters

At T0, conditions were considered as homogeneous in all experimental bags. The statistical analysis showed no significant difference between experimental bags in terms of biological parameters (*i.e.* for bacterial, viral and small eukaryote abundances; mean values are presented in Table 2).

**Table 2:** Initial conditions for chemical and biological parameters

***Chemical and biological parameters in experimental bags at T0***		
	***No nutrient addition***	***+ Nutrient***
PO_4_*μM*	**0.07** (±0.01)	**0.2** (±0.01)
NO_3_*μM*	**0.24** (±0.04)	**0.32** (±0.05)
NH_4_*μM*	**0.48** (±0.04)	**0.44** (±0.005)
NO_2_*μM*	**0.04** (±0.004)	**0.04** (±0.004)
Bacteria 10^6^ cell mL^-1*^	**7.6** (±0.19)	**7.8** (±0.37)
Virus 10^8^ cell mL^-1*^	**1.5** (±0.3)	**1.8** (±0.1)
Picocyanobacteria 10^3^ cell mL^-1*^	**1.4** (±0.09)	**1.5** (±0.06)
Non-pigmented Euk. 10^2^ cell mL^*-1*^	**7.3** (±0.6)	**7.2** (±0.6)
Pigmented Euk. 10^3^ cell mL^*-1*^	**4.3** (±0.6)	**4.4** (±0.6)

Means values (±SD) are presented for the two sets of experimental microcosms (with and without nutrient addition) at T0, for nitrogen and phosphorus compounds, bacteria, viruses, picocyanobacteria, non-pigmented and pigmented small eukaryotes.

* data obtained by flow-cytometry.

#### Abundances and structure of the small eukaryotic community

**Figure 2:**
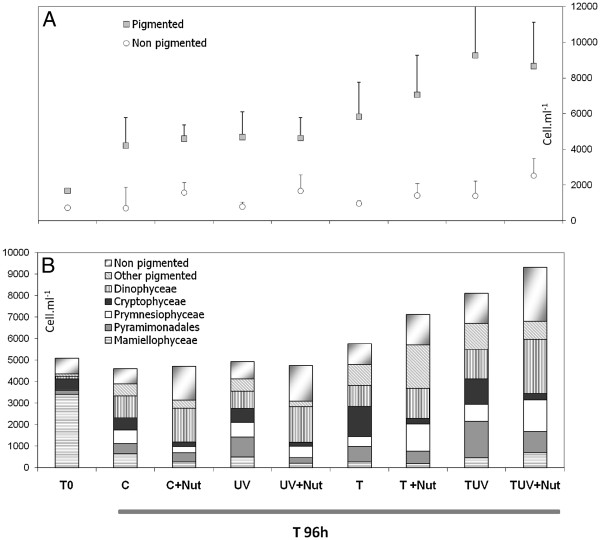
** A. Mean (±SD) abundance of pigmented and non-pigmented small eukaryotes (cell mL**^**-1**^**) at T0 and T96 h in each treatment.** Mean values and SD were calculated from values obtained from treatment triplicates. **B. Relative abundance of different groups identified at T0 and T96 h in each treatment (data obtained from microscopic observation)**.

The microscope counts showed that the eukaryotic community was largely dominated by pigmented cells (85.8% of total eukaryotes). Their mean abundance was 4.3 x10^3^ cells mL^-1^ and 13 of the 26 OTUs identified at T0 from sequencing results were affiliated to pigmented groups (Additional file 2: Table S1). Mamiellophyceae was the dominant group (nearly 83.7% of all pigmented eukaryotes observed by microscopy) and they were represented by 3 OTUs affiliated to *Micromonas pusilla* and *Ostreococcus tauri* (Figure 2 Additional file 2: Table S1). The microscope observations allowed detection of other Viridiplantae at low densities. In particular, some Pyramimonadales (genus *Cymbomonas*) were observed but were not recorded among sequences at T0. The mean relative abundance of Cryptophyceae (4 OTUs) was 10.9%, while very low relative abundances of Bacillariophyceae (1 OTU) and Prymnesiophyceae (represented by *Chrysochromulina-*like cells*,* and 2 OTUs) were found by microscopy (Figure 2) and sequencing. Finally, Dinophyceae (cells larger than 6 μm) accounted for only 3% of total pigmented eukaryotes abundance, and was represented by 1 OTU (Figure 2 Additional file 2: Table S1).

The mean abundance of non-pigmented eukaryotes was 776 cells mL^-1^ at T0, accounting for about 15% of total eukaryotes. In comparison to microscope counting, the proportion of typical non-pigmented eukaryotes was over-estimated in the clone library, accounting for 43.2% of total clones (such over-representation of non-pigmented groups in 18S rRNA gene clone libraries has been discussed previously e.g.[50-52]). The diversity of these non-pigmented groups cannot be discriminated by classical microscopy due to a lack of distinct morphological features and/or their small size. However, from cloning-sequencing results, 11 different OTUs could be attributed to non-pigmented groups: Cercozoa (2 OTUs), Stramenopiles affiliated to Hyphochytrids (1 OTU), Syndiniales affiliated to *Amoebophrya* (2 OTUs), uncultured alveolates (4 OTUs), and Choanoflagellida (2 OTUs) (Figure 2 Additional file 2: Table S1).

### Changes in the small eukaryotes structure under UVBR, temperature and/or nutrient increase

#### Changes in abundances

At T96h, pigmented eukaryotes had abundances varying between 1.1 x10^3^ cells mL^-1^ (C) and 8.3 x10^3^ cells mL^-1^ (TUV) according to the treatment, and they still dominated small eukaryotes regardless of the treatment (Figure 2). All treatments with increased temperature were characterised by a significant increase in the density of pigmented eukaryotes (p < 0.004; Table 3; Figure 2).

**Table 3:** Results of the three-way ANOVA performed from T96h abundance values

**Anova results (P)**	**Temp**	**UV**	**Nut**	**Temp x UV**	**Temp x Nut**	**Temp x UV**	**Temp x UV x Nut**
**Pigmented eukaryotes (total) cells mL**^**-1**^	**0.004 (+)**	NS	NS	NS	NS	NS	NS
Mamiellophyceae	NS	NS	NS	NS	NS	NS	NS
Pyramimonadales	0.059 (+)	0.082 (+)	NS	NS	NS	NS	NS
Prymnesiophyceae	NS	NS	NS	NS	NS	NS	NS
Cryptophyceae	**<0.001 (+)**	NS	**<0.001 (−)**	NS	**0.002**	NS	NS
Bacillariophyceae	NS	NS	NS	NS	NS	NS	NS
Dinophyceae	NS	NS	**0.028 (+)**	NS	NS	NS	NS
**Non-pigmented eukaryotes cells mL**^**-1**^	NS	NS	NS	NS	NS	NS	NS
**Bacteria cell mL**^**-1**^	**<0.001 (+)**	**0.013 (−)**	NS	NS	NS	NS	NS
**Virus particles mL**^**-1**^	**0.008(+)**	**<0.001 (−)**	NS	**0.001**	NS	NS	NS
**Picocyanobacteria cells mL**^**-1**^	NS	NS	**<0.001 (+)**	NS	NS	NS	**0.013**

P values obtained for the effects of temperature (Temp), UVBR (UV), nutrient addition (Nut) and the interactions between the three factors are presented. + and – signs indicate the direction of the effect (positive or negative impact). Bold font corresponds to significant values, where p < 0.05, while normal font corresponds to a lower significance (p < 0.1). NS is the code for a non-significant effect.

Some major changes were observed in the relative proportions of the main taxonomic groups. The abundance of pigmented Dinophyceae increased in all treatments, with the highest increases where nutrients were added. Indeed, the 3-way ANOVA showed a significant effect of nutrients (p = 0.028, Table 3). Inversely, for Cryptophyceae, a general negative impact of nutrient addition (p < 0.001) counteracted the positive impact of temperature increase (Table 3, Figure 2). The relative abundance of Mamiellophyceae (*Micromonas* and *Ostreococcus)* decreased from T0 to T96h in all treatments, and they represented only between 0.1 and 14.8% of pigmented eukaryotes at the end of the experiment (depending on the treatment). Pyramimonadales seemed to take advantage of the general reduction of Mamiellophyceae densities and developed strongly, especially in treatments with increased UVBR. The 3-way ANOVA showed a positive impact of UVBR on Pyramimonadales abundance.

Non-pigmented eukaryotes (mainly free flagellated forms) tended to increase in abundance in all conditions. The highest values were found in TUV + Nut treatments (mean abundance: 2.5 x10^3^ cells mL^-1^), however, the 3-way ANOVA did not reveal any significant impact of the manipulated factors (Table 3).

#### Changes in small eukaryotes structure (CE SSCP)

**Figure 3:**
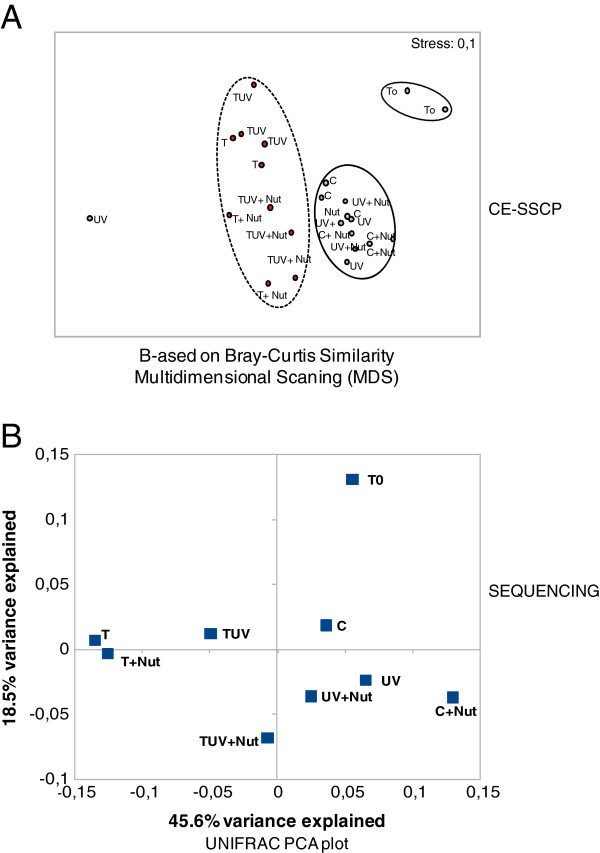
** A. Comparison of diversity profiles obtained by CE-SSCP (based on Bray-Curtis Similarity).** Replicates were analysed separately. **B. UNIFRAC analysis comparing the composition (representation of OTUs) of the nine clone libraries** (one library at T0 and eight at T96h). Treatment triplicates were pooled.

A Multidimensional Scaling (MDS) plot generated from Bray-Curtis similarity indices based on the 18S rDNA CE-SSCP profiles, showed that all samples from temperature increase simulation (treatments T, T + Nut, TUV, TUV + Nut) grouped in a separate cluster (Figure 3A). The small eukaryotic community structures of all other treatments (without temperature increase) had closer similarity to initial conditions. Overall, CE-SSCP profiles generated from all experimental bags showed good reproducibility within triplicate of each treatment (ANOSIM R < 0.2, p < 0.001), except for one replicate of the UVBR condition which had an atypical profile. MDS ordination plot stress value was low (0.1) which indicated good ordination without misleading interpretation [53]. The same trends were found with the UPGMA (Unweighted Pair Group Method using Arithmetic averages) analysis (data not shown).

#### Changes in small eukaryotes phylogenetic composition (sequencing)

**Figure 4:**
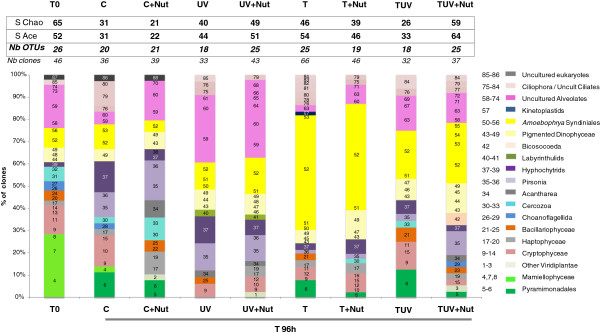
** Composition of the nine 18SrRNA gene clone libraries.** Each histogram represents the community structure expressed by the diversity of OTUs (a total of 88 OTUs were detected), their affiliation to phylogenetic groups, and the proportion of clones per OTU. The S_ACE_ and S_Chao1_ value (richness estimators) and number of OTUs are specified on the top of each histogram. Arbitrarily assigned OTU reference numbers are given in each section of the histogram, and their taxonomic affiliations are presented in the key.

A total of 88 OTUs were identified (97% similarity) (Additional file 2: Table S1; and phylogenetic tree in Additional file 1: Figure S1)*.* During the incubation, the richness detected by molecular analyses showed a general decrease in 7 (out of the 8) treatments (Figure 4). TUV + Nut was the only treatment characterised by a clear increase in the richness (SAce = 64), whereas the greatest decrease was recorded in the C + Nut treatment (SAce = 22). Even though no general trend was observed in the responses of small eukaryotes in terms of overall richness, the beta-diversity (phylogenetic composition) studied from UNIFRAC metrics revealed a clear association between all treatments with increased temperature (discrimination on axis 1). This highlights the significant structuring impact of increased temperature, while on axis 2, nutrient addition appeared as the second-most important factor in shaping the eukaryotic composition (Figure 3B). These observations were confirmed by analyzing the correlations between coordinates on the PCA axis and environmental parameters: coordinates on axis 1 were indeed significantly correlated to temperature values (P = 0.006) while coordinates on axis 2 were significantly correlated to inorganic nutrients concentrations (P = 0.046 and P = 0.006, respectively for NO_2_ and NO_3_). The P-values matrix that compares each sample to each other sample showed significant differences in the phylogenetic composition of eukaryotes between T, T + Nut, TUV on the one hand and C + Nut on the other (Additional file 2: Table S2). Thus, CE-SSCP profiles and UNIFRAC analysis led to the same general pattern of changes in the small eukaryote structure.

The OTUs affiliated to non-pigmented taxa generally dominated the clone libraries (from 67.6% in C + Nut to 85.3% in UV + Nut; Figure 4 and Additional file 2: Table S1). Among them, Ciliates and uncultured Alveolates were generally well represented (accounting from 14 to 32% of total OTUs, and from 13 to 37% of clones, according to the treatments). However, the increase of non-pigmented group proportions within most of the libraries (compared to T0) was mainly linked to the emergence of taxa affiliated to parasitic groups: Hyphochytrids and genus *Pirsonia* (Heterokonta), and *Amoebophrya* (Alveolata).

**Figure 5:**
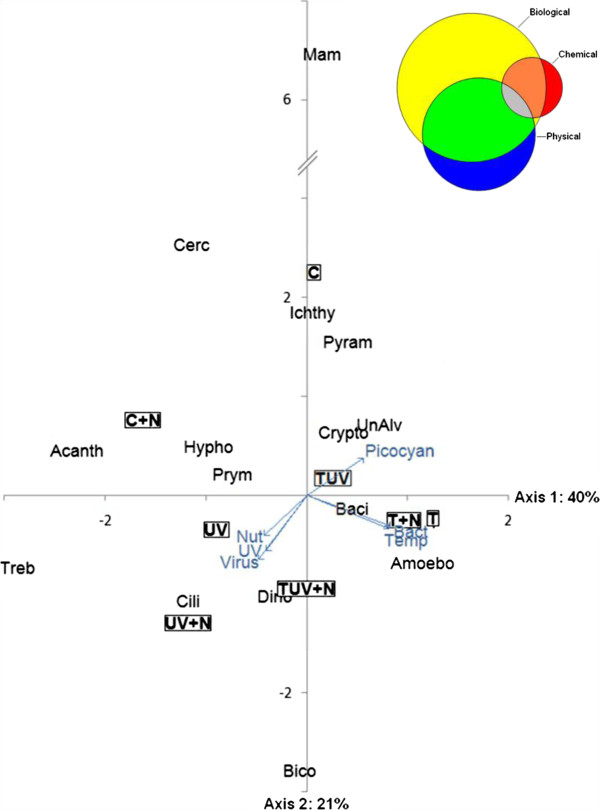
**Correspondence Canonical Analysis (CCA) performed on the sequencing results expressed as proportion of OTUs detected in the eight libraries constructed at T96 h (i.e. C, UV, T, TUV, C + N, UV + N, T + N, TUV + N treatments).** Environmental variables are heterotrophic bacteria (Bact), picocynobacteria (Picocyan), viruses (virus), temperature (Temp), UVB radiation (UV), nutrient concentration (Nut). Phylogenetic groups are Trebouxiophyceae (Treb), Pyramonadales (Pyram), Mamiellophyceae (Mam), Cryptophyceae (Crypto), Prymnesiophyceae (Prym), Bacillariophyceae (Baci), pigmented Dinophyceae (Dino), Ichthyosporea (Ichthy), Cercozoa (Cerc), Acantharea (Acanth), Hyphochytrids (Hypho), Bicosoecida (Bico), Amoebophrya (Amoebo), Ciliates (Cili), Uncultured Alveolates (UnAlv). The Venn diagram illustrates the relative proportions of the variation in sequence data that could be associated with variation in biological, chemical and physical parameters from the eigenvalues calculated by the CCA.

The proportion of these sequences clearly increased during the incubation in all types of treatment. Parasitic taxa related to *Amoebophrya* particularly emerged in treatments with the highest temperatures (T, T + Nut, TUV, and to a lesser extent TUV + Nut), while Hyphochytrids were strongly associated with all other treatments (C, C + Nut, UV, UV + Nut) (Figure 4). The CCA plot illustrates the significant link between the increase in temperature and the presence of numerous sequences affiliated to *Amoebophrya*, while sequences affiliated to Hyphochytrides have an opposite position in the plot (Figure 5). The potential hosts of *Amoebophrya* are primarily found within the class Dinophyceae, and it is noticeable that we observed a large number of pigmented Dinophyceae cells infected by parasites (multinucleated parasites in division in the cells) at T96 h in all types of treatment (data not shown). Pigmented Dinophyceae were indeed favored by the temperature increase but were also strongly positively affected by nutrient addition and UVBR increase (Figure 5). Pigmented Dinophyceae and *Amoebophrya* were represented by 7 different OTUs each. Even though the presence/absence of these OTUs varied according to the treatments, no association between the abundance of host and parasite OTUs was observed.

The CCA supported the conclusion obtained from the UNIFRAC analysis, clearly showing that all treatments with increased temperature grouped together. Furthermore, the highest abundances of bacteria, picocyanobacteria, and pigmented groups such as Cryptophyceae and Bacillariophyceae were tightly associated with treatments receiving an increased temperature (Figure 5).

The CCA plot also illustrates the strong negative impact of experimental conditions on Mamiellophyceae in general. Mamiellophyceae represented 28% of sequences in the clone library at T0, but were not detected at T96 h (except 1 OTU detected in the C treatments). In contrast, Pyramimonadales sequences (2 OTUs) appeared at T96 h in 6 out of the 8 types of treatment.

Overall, the analysis of the OTUs dynamics (either generally or for specific phylogenetic groups) showed that, even when the abundance of a given group did not change significantly from one treatment to another, some rearrangements could occur at the OTUs level (Additional file 2: Table S1). The CCA showed that 18.8% of the total variation in the eukaryotic structure was explained by temperature, whereas, UVBR and nutrients explained 11% and 8.4%, respectively.

## Discussion

The Thau lagoon, characterised by a high abundance of small eukaryotes and by recent *in situ* changes in phytoplankton structure due to water temperature increase [27], is an interesting ecosystem to investigate the responses of small eukaryotes to climatic and anthropogenic regulatory factors. Our experimentation does not intend to predict the impact of long-term global change on the structure of small planktonic eukaryotes. Indeed, only a combination of approaches including laboratory studies on model microbes, microcosm and mesocosm experiments, and *in situ* comparative studies would help to provide realistic predictions of the effects of environmental changes [23,54]. Our goal was to reveal the potential rapid responses of small eukaryote assemblage (using molecular and morphological methods) during the productive spring season when plankton may be particularly vulnerable to elevated temperature and UVBR [55].

Molecular analyses revealed the presence of various phylogenetic groups within the “black box” of small eukaryotes, especially non-pigmented eukaryotes (poorly discriminated by microscopy). Some limitations in the PCR-based methods are recognized, for instance, the over-representation of Alveolata (particularly Dinoflagellates and Ciliates) in 18S rRNA gene clone libraries due their high SSU rRNA gene copy number [50-52]. However, the molecular methods used here enabled the description of the diversity within dominant eukaryotic populations, and allowed examination of the effects of regulatory factors by considering both the dynamics of OTUs (using the sequencing and fingerprinting datasets) and the comparison of phylogenetic composition obtained for all treatments (using the sequencing data). The impact of temperature nutrients and UVBR explained 18.8%, 11.0% and 8.4% of the variance of the small eukaryotes structure respectively. While Bouvy *et al*. (2011) could not detect any significant responses of pico- or nano-eukaryotic plankton in the same experimental conditions, we demonstrated here, at a different taxonomic resolution, that small eukaryotes community structure was actually affected by this multi-factorial pressure.

The simultaneous use of molecular and morphological methods was therefore essential to provide evidence of rapid shifts that occur at various taxonomic levels (abundance of large groups or community composition at OTU level) under the influence of temperature, UVBR and nutrient treatments.

Among the 3 regulatory factors tested, both sequencing and CE-SSCP demonstrated that increased temperature had the greatest influence on the small eukaryote community structure and composition. The single effect of temperature (without any significant interaction with UVBR and nutrients) on total pigmented eukaryote abundance was observed by microscopy. Considering the different phylogenetic groups within pigmented eukaryotes, complex interaction effects were also suggested. For instance, our results showed that under multi-factorial environmental changes, the general impact on the molecular diversity and abundance of pigmented Dinophyceae resulted from complex interactive (non-additive) effects. Multi-factorial interactions were also apparent for Cryptophyceae which experienced antagonistic effects of nutrient addition (significantly negative impact) and temperature (positive impact on relative abundance).

In addition to the manipulated factors (temperature, UVBR and nutrients), some biotic interactions such as predation, viral lysis and competition, are involved in the responses observed in this experiment. For example, the general reduction of Mamiellophyceae (*Micromonas* and *Ostreococcus*) in all treatments might be linked to (i) manipulation effects since these fragile cells might have been affected by filtration steps, (ii) limitation by inorganic nutrients under the rather low orthophosphate concentrations at T96h (from 0.05 to 0.08 μM of PO_4_), (iii) the grazing impact of heterotrophic flagellates: these microorganisms are known to play a significant role in the regulation of *Ostreococcus* populations in the Thau lagoon [56] and were shown to exert a strong control of bacterioplankton during the study period [24]. We could not detect a link between the dynamics of *Micromonas*/*Ostreococcus* and viruses. Since biological descriptors can explain some of the variance of small eukaryote phylogenetic structure, the observations made here regarding the effect of temperature, UVBR, and nutrients have to be considered in view of possible biological effects. Predation by zooplankton and competition with larger phytoplanktonic species were not considered in our size fractionated approach and should be taken into account, especially if long-term extrapolation of *in situ* responses of small eukaryotes is considered.

Our data provide further illustration of the need to consider the taxonomic and functional diversity of heterotrophic flagellates. The lack of discrimination between heterotrophic bacterivores and parasitic/saprotrophic zoospores within the non-pigmented flagellates can lead to misinterpretation of the functioning and responses of planktonic food webs. Indeed, while microscope observations did not allow us to detect changes in the abundance and structure of non-pigmented eukaryotes, a structuring impact of manipulated factors (especially temperature) was observed through sequencing results on taxa affiliated to parasitic and saprotroph groups (particularly Syndiniales and Hyphochytrids). The existence of eukaryotic parasites among small-size plankton was recently re-discovered by molecular environmental surveys, and the ecological significance of these groups has been highlighted by several authors [57,58]. The ‘Fungi-like’ Hyphochytrids possess many morphological and ecological similarities to chytrids [58,59], and their role as saprotrophs and/or parasites is unclear [60,61], whereas the *Amoebophrya* are well recognized as a widely distributed parasitic order within the Dinophyceae [62]. *Amoebophrya* and Hyphochytrids emerged in clone libraries at T96 h and were presumably present among the rare species at T0. The taxa found to be phylogenetically close to *Amoebophrya* particularly emerged in treatments with increased temperature (Figure 5), along with their hosts (pigmented Dinoflagellates). This observation supports Guillou *et al.*’s [57] suggestion that warming could promote rapid infection cycles of *Amoebophrya.* However, broad extrapolation would need to take into account various aspects of the host-parasite relationships, such as the mechanisms underlying the parasitic specificity. In contrast to the *Amoebophrya*, hyphochytrids were associated with all treatments except those with increased temperature (Figure 5). From our results, we hypothesized that not only parasite communities, but also saprotroph communities would be shaped by temperature and UVBR conditions, as already described in other ecosystems [63]. The responses of saprotrophs to these drivers may result from direct and/or indirect effects as demonstrated in soils [64]; further research is probably needed on the saprotrophs in aquatic systems since changes in their assemblages may influence organic matter decomposition and nutrient cycling.

## Conclusion

Even though caution should be exercised when applying the results of small-scale experiments to larger-scale systems, these results can be treated as an insight into ecological interactions that may occur in larger natural systems with more complex planktonic assemblages. Our results indicate that these ecosystem drivers, which are associated with climate change, and their interactions may cause changes in small eukaryotic community abundance and structure involving various functional groups including the small primary producers, parasites and saprotrophs. Notably, temperature tends to have a much greater effect on the community composition of small eukaryotes compared to UVBR (at least at the level tested in our experiment). Due to their strong link with other communities within the food web, the small eukaryotes variability may have potential consequences in food webs structure and energy flow. Currently, our knowledge of the potential for plankton in general and small eukaryotes in particular to adapt genetically and phenotypically to multifactorial physico-chemical climate drivers is poor. To improve our understanding, additional experimental investigations in other types of ecosystems and over longer periods of warming and UVBR exposure are required before generalization may be confidently applied. Future investigations should be based on the coupling of methods such as microscopy, flow cytometry, molecular analyses targeting several gene markers or fluorescence *in situ* hybridization in order to analyse the responses of the microbial community structure to multiple stressors at various taxonomic levels.

## Authors’ contributions

All authors have made substantial intellectual contributions to the study. They read and approved the final manuscript. TB was the principal investigator of this study. TB, ID, MB, SJ, JPT, YB, FV, BM, EL, EF participated in the experimental design. BM, EL, TB supervised the operational realisation of the experiment. ID, HM, CB, EF, EL realised chemical (nutrients) and biological analyses (microscopic observations), SJ performed the flow cytometric analysis. JFG performed and interpreted the CE-SSCP analysis. CL, ID, DD performed the molecular analyses and the post sequencing analysis, AK contributed with CL ID and DD to the statistical analysis. Writing was mainly prepared by ID, CL, DD and MB, helped by AK, JFG, SJ, FV, BM, YB, JPT, TB.

## Supplementary Material

Additional file 1 ** Figure S1. Maximum parsimony tree showing phylogenetic relationships of the partial 18S rRNA gene sequences.** The tree was constructed with the 376 sequences generated in this study and sequences from genbank. Only one representative sequence per OTU per library is presented in this phylogenetic tree. The labels show the origin of each sequence (treatments: C, C+Nut, UV, UV+Nut, T, T+Nut, TUV, TUV+Nut, and, time: T0 and T96 h). Values in brackets correspond to the OTU numbers as presented in Figure 4 and Additional file 2: Table S1. Click here for file

Additional file 2 ** Table S1. Composition of the nine 18S rRNA genes clone libraries in terms of OTUs at T0 and T96h, the affiliation to phylogenetic groups is specified for each OTU.** * The number associated to each OTU corresponds to numbers used in Figure 4 and in the phylogenetic tree (Additional file 1: Figure S1). **Table S2. UNIFRAC metrics:** The grey area (right panel) corresponds to the distance matrix obtained from the comparison of each pair of samples. Bold text denotes values in the upper quartile (i.e. most distant samples). The white area (left panel) corresponds to the P-values obtained by comparing each sample to each other sample. All P-values have been corrected for multiple comparisons by multiplying the calculated P-value by the number of comparisons made (Bonferroni correction). Bold text denotes significant P values. Click here for file
