# Supplementary material for: Short-term responses of unicellular planktonic eukaryotes to increases in temperature and UVB radiation
Source: BMC Microbiol. 2012 Sep 11;12:202. doi: 10.1186/1471-2180-12-202 (PMC3478981; doi:10.1186/1471-2180-12-202)
Supplement: Additional file 1 — Figure S1. Maximum parsimony tree showing phylogenetic relationships of the partial 18S rRNA gene sequences. The tree was constructed with the 376 sequences generated in this study and sequences from genbank. Only one representative sequence per OTU per library is presented in this phylogenetic tree. The labels show the origin of each sequence (treatments: C, C+Nut, UV, UV+Nut, T, T+Nut, TUV, TUV+Nut, and, time: T0 and T96 h). Values in brackets correspond to the OTU numbers as presented in Figure 4 and Additional file 2: Table S1. [file 1471-2180-12-202-S1.pdf]

SUPLEMENTAL DATA

Four enclosures (polyethylene bags, 1.2 m diameter x 3 m depth) acted as incubators for the Whirl-Pak bags (2 L experimental bags). The four simulated climatic conditions were: (1) *in situ* temperature and UV-B radiation, (2) + 3 °C , (3) +20% UVBR, (4) +3 °C and +20% UVBR. The regulation of UVBR and temperature was achieved with a high frequency monitoring of the *in situ* temperature, the natural incident UVBR and reproducing the natural solar spectrum.

The increased UVBR levels were achieved using 2 UVB lamps (Philips TL20RS/01 - emission peak 312 nm) regulated by electronic ballasts and controlled by a central Data Logger (Campbell Scientific CR23X). The light intensity of lamps gave an increase of 20% in comparison to UVB incident light measured by a UVB reference captor (Skye, SKU 430) situated near the enclosures.

The Whirl-Pak bags actually transmitted only 70% of total UV light. Consequently in these experimental bags (located at subsurface) the planktonic communities were exposed to UVBR that represent 84% of the *in situ* UV radiation. Nonetheless, the treatments with an UVBR increase received actually +20% UVBR compared to the other treatments, and therefore the between-treatment differential of 20% UV remains valid.

It is important to mention that the amount of UVBR received by planktonic communities in the experimental bags corresponds to a realistic situation if we consider *in situ* conditions at a depth of 40 cm of the water column: the studied lagoon has a mean depth of 4.5 m, organisms in natural mixed conditions are obviously expected to receive less UVB than at the subsurface. As the depth at which 1% of UVB incident penetrated was around 1.4 m [22] and as light is attenuated in an exponential manner, 70 % of incident light corresponds to a depth of 40 cm.

The increased temperature was achieved with a submersible heating element (Galvatec, France). Similarly to the increased UVBR levels, the temperature augmentation (+3 °C) was kept constant using a control station Campbell Scientific which measures *in situ* temperature every 30 seconds.

Each enclosure was equipped with 3 measuring probes so that temperature (surface, middle, bottom of the enclosure) could be continuously monitored and with a water pump which ensure the mixing and the homogenisation of water mass.

This experimental facility allowed us to reproduce the nycthemeral variations of UVBR and *in situ* temperature faithfully. A fully description of MEDIMEER infrastructures facilities (i.e. additional details on the enclosure set up and the automatic closed loop regulation system controlling water temperature and UVBR enhancements) is provided in two previous publications [22, 25].

Table S1. Composition of the nine 18S rRNA genes clone libraries in terms of OTUs at T0 and T96h, the affiliation to phylogenetic groups is specified for each OTU.

* The number associated to each OTU corresponds to numbers used in Figure 4 and in the phylogenetic tree (FigS1)

	N°OTU *	T0	T 96h							
			C	C+Nut	UV	UV+Nut	T	T+Nut	TUV	TUV+Nut
Pyramimonadales	5			1						1
	6		4	2			5	1	4	
Mamiellophyceae	4	7	1							
	7	5								
	8	1								
Other Viridiplantae	1					1				
	2			1						
	3									1
Cryptophyceae	9	2	1		2	1	1		2	
	10		3			1		1		
	12					1	2	1		
	15		1					1	1	1
	16							1		
	11	2					1		1	
	13	1								
	14	1								
Haptophyceae	17	1	1	2		1	1	2		
	18						1			
	19			2		1				1
	20	1								
Bacillariophyceae	21						2		2	
	22			1						
	23									1
	24	1								
	25			1	1					
Choanoflagellida	26	1								
	27	1								
	28		1							
	29									1
Cercozoa	30		1	2				1		
	31	2								
	32	1								
	33			2					1	
Acantharea	34			3	1	1				1
Pirsonia	35		3	5	4	4		1	1	4
	36		1	2			1			
Hyphochytrids	37		5	1	4	3	2	3	2	1
	38			1						
	39	1								
Labyrinthulida	40				1					
	41					1				
Bicosoecida	42									2
Dinophyceae (pigmented)	43			2	1		1	1	1	1
	44	1			1					2
	45						1			1
	46					1			1	
	47					1		1	1	
	48	1				1				
	49	1	2	1	1	1	2	4		1
Dinophyceae / Amoebophrya	50				1		1			
	51				1	1	2	1	1	
	52	3	2	2	2	6	30	21	6	6
	53		2				1			1
	54									
	55									1
	56	1								
Kinetoplastids	57						1			
Uncultured Aveolates	58	2								1
	59	5	1	3	7	5				
	60		1	3	2	3		1		
	61				1					
	62					1				
	63						2	2	3	2
	64					2				
	65					1				
	66					1				
	67								1	
	68					2				
	69								1	
	70			1						
	71							1		1
	72									1
	73	1								
	74	1								
Ciliophora Uncultured ciliates	75				1			1		
	76		1		1		1		1	
	77									1
	78						1			
	79		3			1		1		1
	80		1				2			
	81						2			
	82						1			
	83						1			
	84						1		2	1
Uncultured eukaryotes	85	1			1					
	86		1							
	87	1								
	88			1						

Table S2: UNIFRAC metrics**:**
The grey area (right panel) corresponds to the distance matrix obtained from the comparison of each pair of samples. Bold text denotes values in the upper quartile (i.e. most distant samples).
The white area (left panel) corresponds to the P-values obtained by comparing each sample to each other sample. All P-values have been corrected for multiple comparisons by multiplying the calculated P-value by the number of comparisons made (Bonferroni correction). Bold text denotes significant P values.

	T0	UV	UV+Nut	C	C+Nut	T	T+Nut	TUV	TUV+Nut
T0		0.180	0.183	0.157	**0.210**	**0.236**	**0.237**	0.194	**0.216**
UV	0.59		0.094	0.126	0.168	**0.222**	**0.221**	0.182	0.153
UV+Nut	1.00	1.00		0.136	0.178	0.193	0.181	0.158	0.123
C	0.70	0.90	0.94		0.167	0.194	0.192	0.159	0.152
C+Nut	0.31	0.26	0.68	0.84		**0.280**	**0.269**	**0.230**	0.196
T	0.14	0.90	0.78	0.88	**0.03****		0.076	0.151	0.180
T+Nut	0.85	0.68	1.00	1.00	**0.09***	0.97		0.164	0.181
TUV	0.95	1.00	1.00	0.99	**0.10***	0.96	1.00		0.149
TUV+Nut	0.95	1.00	1.00	0.99	**0.13**	0.96	1.00	0.99	
