# Supplementary material for: Short-term responses of unicellular planktonic eukaryotes to increases in temperature and UVB radiation
Source: BMC Microbiol. 2012 Sep 11;12:202. doi: 10.1186/1471-2180-12-202 (PMC3478981; doi:10.1186/1471-2180-12-202)
Supplement: Additional file 2 — Table S1. Composition of the nine 18S rRNA genes clone libraries in terms of OTUs at T0 and T96h, the affiliation to phylogenetic groups is specified for each OTU. * The number associated to each OTU corresponds to numbers used in Figure 4 and in the phylogenetic tree (Additional file 1: Figure S1). Table S2. UNIFRAC metrics: The grey area (right panel) corresponds to the distance matrix obtained from the comparison of each pair of samples. Bold text denotes values in the upper quartile (i.e. most distant samples). The white area (left panel) corresponds to the P-values obtained by comparing each sample to each other sample. All P-values have been corrected for multiple comparisons by multiplying the calculated P-value by the number of comparisons made (Bonferroni correction). Bold text denotes significant P values. [file 1471-2180-12-202-S2.pdf]

Figure S1.

Maximum parsimony tree showing phylogenetic relationships of the partial 18S rRNA gene sequences. The tree was constructed with the 376 sequences generated in this study and sequences from genbank.

Only one representative sequence per OTU per library is presented in this phylogenetic tree. The labels show the origin of each sequence (treatments: C, C+Nut, UV, UV+Nut, T, T+Nut, TUV, TUV+Nut, and, time: T0 and T96 h). Values in brackets correspond to the OTU numbers as presented in Fig.4 and table S1.

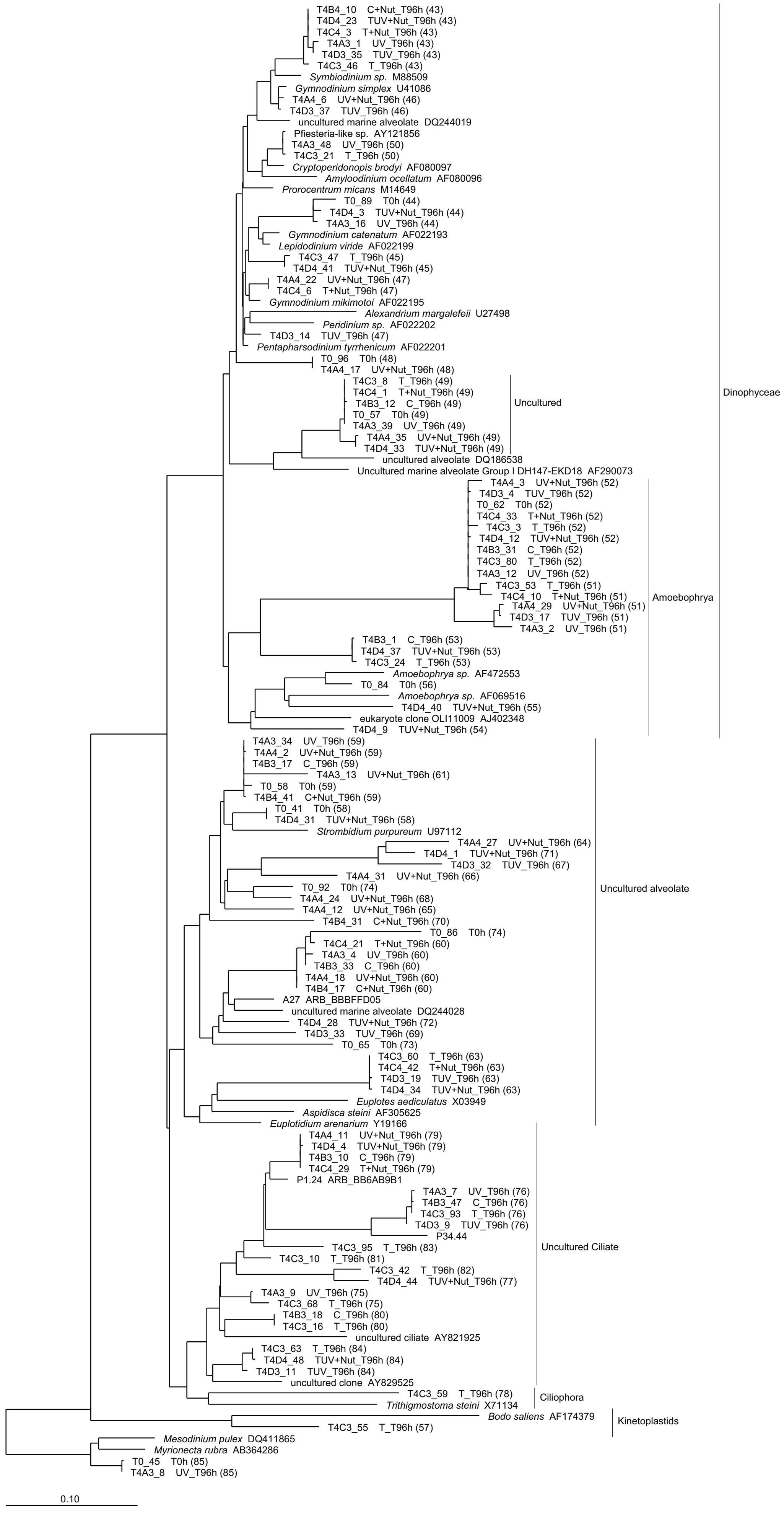

T4B4_10 C+Nut_T96h (43)
T4D4_23 TUV+Nut_T96h (43)
T4C4_3 T+Nut_T96h (43)
T4A3_1 UV_T96h (43)
T4D3_35 TUV_T96h (43)
T4C3_46 T_T96h (43)
Symbiodinium sp. M88509
Gymnodinium simplex U41086
T4A4_6 UV+Nut_T96h (46)
T4D3_37 TUV_T96h (46)
uncultured marine alveolate DQ244019
Pfiesteria-like sp. AY121856
T4A3_48 UV_T96h (50)
T4C3_21 T_T96h (50)
Cryptoperidonopis brodyi AF080097
Amyloodinium ocellatum AF080096
Prorocentrum micans M14649
T0_89 T0h (44)
T4D4_3 TUV+Nut_T96h (44)
T4A3_16 UV_T96h (44)
Gymnodinium catenatum AF022193
Lepidodinium viride AF022199
T4C3_47 T_T96h (45)
T4D4_41 TUV+Nut_T96h (45)
T4A4_22 UV+Nut_T96h (47)
T4C4_6 T+Nut_T96h (47)
Gymnodinium mikimotoi AF022195
Alexandrium margalefeii U27498
Peridinium sp. AF022202
T4D3_14 TUV_T96h (47)
Pentapharsodinium tyrrhenicum AF022201
T0_96 T0h (48)
T4A4_17 UV+Nut_T96h (48)
T4C3_8 T_T96h (49)
T4C4_1 T+Nut_T96h (49)
T4B3_12 C_T96h (49)
T0_57 T0h (49)
T4A3_39 UV_T96h (49)
T4A4_35 UV+Nut_T96h (49)
T4D4_33 TUV+Nut_T96h (49)
uncultured alveolate DQ186538
Uncultured marine alveolate Group I DH147-EKD18 AF290073
Uncultured
T4A4_3 UV+Nut_T96h (52)
T4D3_4 TUV_T96h (52)
T0_62 T0h (52)
T4C4_33 T+Nut_T96h (52)
T4C3_3 T_T96h (52)
T4D4_12 TUV+Nut_T96h (52)
T4B3_31 C_T96h (52)
T4C3_80 T_T96h (52)
T4A3_12 UV_T96h (52)
T4C3_53 T_T96h (51)
T4C4_10 T+Nut_T96h (51)
T4A4_29 UV+Nut_T96h (51)
T4D3_17 TUV_T96h (51)
T4A3_2 UV_T96h (51)
T4B3_1 C_T96h (53)
T4D4_37 TUV+Nut_T96h (53)
T4C3_24 T_T96h (53)
Amoebophrya sp. AF472553
T0_84 T0h (56)
Amoebophrya sp. AF069516
T4D4_40 TUV+Nut_T96h (55)
eukaryote clone OLI11009 AJ402348
T4D4_9 TUV+Nut_T96h (54)
Amoebophrya
Dinophyceae
T4A3_34 UV_T96h (59)
T4A4_2 UV+Nut_T96h (59)
T4B3_17 C_T96h (59)
T4A3_13 UV+Nut_T96h (61)
T0_58 T0h (59)
T4B4_41 C+Nut_T96h (59)
T0_41 T0h (58)
T4D4_31 TUV+Nut_T96h (58)
Strombidium purpureum U97112
T4A4_27 UV+Nut_T96h (64)
T4D4_1 TUV+Nut_T96h (71)
T4D3_32 TUV_T96h (67)
T4A4_31 UV+Nut_T96h (66)
T0_92 T0h (74)
T4A4_24 UV+Nut_T96h (68)
T4A4_12 UV+Nut_T96h (65)
T4B4_31 C+Nut_T96h (70)
T0_86 T0h (74)
T4C4_21 T+Nut_T96h (60)
T4A3_4 UV_T96h (60)
T4B3_33 C_T96h (60)
T4A4_18 UV+Nut_T96h (60)
T4B4_17 C+Nut_T96h (60)
A27 ARB_BBBFFD05
uncultured marine alveolate DQ244028
T4D4_28 TUV+Nut_T96h (72)
T4D3_33 TUV_T96h (69)
T0_65 T0h (73)
T4C3_60 T_T96h (63)
T4C4_42 T+Nut_T96h (63)
T4D3_19 TUV_T96h (63)
T4D4_34 TUV+Nut_T96h (63)
Euplotes aediculatus X03949
Aspidisca steini AF305625
Euplotidium arenarium Y19166
Uncultured alveolate
T4A4_11 UV+Nut_T96h (79)
T4D4_4 TUV+Nut_T96h (79)
T4B3_10 C_T96h (79)
T4C4_29 T+Nut_T96h (79)
P1.24 ARB_BB6AB9B1
T4A3_7 UV_T96h (76)
T4B3_47 C_T96h (76)
T4C3_93 T_T96h (76)
T4D3_9 TUV_T96h (76)
P34.44
T4C3_95 T_T96h (83)
T4C3_10 T_T96h (81)
T4C3_42 T_T96h (82)
T4D4_44 TUV+Nut_T96h (77)
T4A3_9 UV_T96h (75)
T4C3_68 T_T96h (75)
T4B3_18 C_T96h (80)
T4C3_16 T_T96h (80)
uncultured ciliate AY821925
T4C3_63 T_T96h (84)
T4D4_48 TUV+Nut_T96h (84)
T4D3_11 TUV_T96h (84)
uncultured clone AY829525
Uncultured Ciliate
T4C3_59 T_T96h (78)
Trithigmostoma steini X71134
Ciliophora
Bodo saliens AF174379
T4C3_55 T_T96h (57)
Kinetoplastids
Mesodinium pulex DQ411865
Myrionecta rubra AB364286
T0_45 T0h (85)
T4A3_8 UV_T96h (85)
0.10

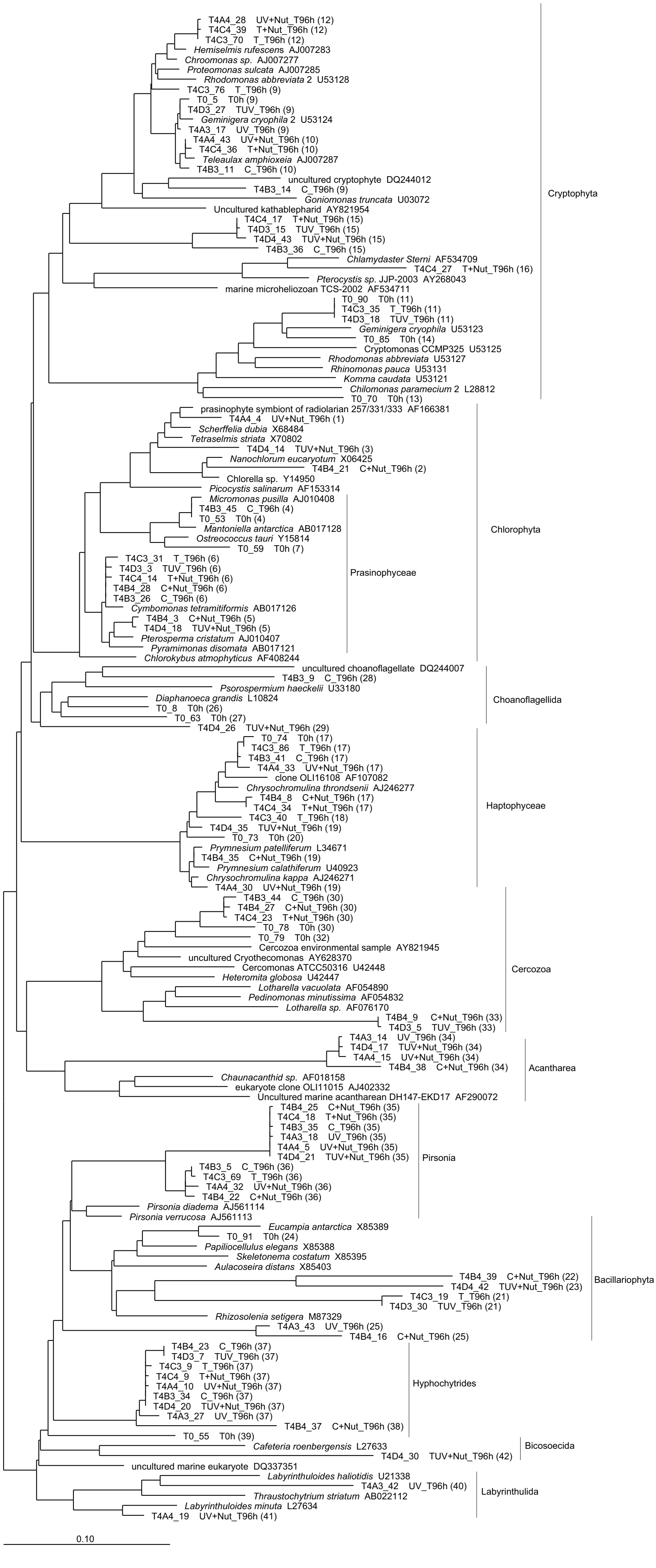

T4A4_28 UV+Nut_T96h (12)
T4C4_39 T+Nut_T96h (12)
T4C3_70 T_T96h (12)
Hemiselmis rufescens AJ007283
Chroomonas sp. AJ007277
Proteomonas sulcata AJ007285
Rhodomonas abbreviata 2 U53128
T4C3_76 T_T96h (9)
T0_5 T0h (9)
T4D3_27 TUV_T96h (9)
Geminigera cryophila 2 U53124
T4A3_17 UV_T96h (9)
T4A4_43 UV+Nut_T96h (10)
T4C4_36 T+Nut_T96h (10)
Teleaulax amphioxeia AJ007287
T4B3_11 C_T96h (10)
uncultured cryptophyte DQ244012
T4B3_14 C_T96h (9)
Goniomonas truncata U03072
Uncultured kathablepharid AY821954
T4C4_17 T+Nut_T96h (15)
T4D3_15 TUV_T96h (15)
T4D4_43 TUV+Nut_T96h (15)
T4B3_36 C_T96h (15)
Chlamydaster Sterni AF534709
T4C4_27 T+Nut_T96h (16)
Pterocystis sp. JJP-2003 AY268043
marine microheliozoan TCS-2002 AF534711
T0_90 T0h (11)
T4C3_35 T_T96h (11)
T4D3_18 TUV_T96h (11)
Geminigera cryophila U53123
T0_85 T0h (14)
Cryptomonas CCMP325 U53125
Rhodomonas abbreviata U53127
Rhinomonas pauca U53131
Komma caudata U53121
Chilomonas paramecium 2 L28812
T0_70 T0h (13)
Cryptophyta
prasinophyte symbiont of radiolarian 257/331/333 AF166381
T4A4_4 UV+Nut_T96h (1)
Scherffelia dubia X68484
Tetraselmis striata X70802
T4D4_14 TUV+Nut_T96h (3)
Nanochlorum eucaryotum X06425
T4B4_21 C+Nut_T96h (2)
Chlorella sp. Y14950
Picocystis salinarum AF153314
Micromonas pusilla AJ010408
T4B3_45 C_T96h (4)
T0_53 T0h (4)
Mantoniella antarctica AB017128
Ostreococcus tauri Y15814
T0_59 T0h (7)
T4C3_31 T_T96h (6)
T4D3_3 TUV_T96h (6)
T4C4_14 T+Nut_T96h (6)
T4B4_28 C+Nut_T96h (6)
T4B3_26 C_T96h (6)
Cymbomonas tetramitiformis AB017126
T4B4_3 C+Nut_T96h (5)
T4D4_18 TUV+Nut_T96h (5)
Pterosperma cristatum AJ010407
Pyramimonas disomata AB017121
Chlorokybus atmophyticus AF408244
Chlorophyta
Prasinophyceae
uncultured choanoflagellate DQ244007
T4B3_9 C_T96h (28)
Psorospermium haeckelii U33180
Diaphanoeca grandis L10824
T0_8 T0h (26)
T0_63 T0h (27)
T4D4_26 TUV+Nut_T96h (29)
Choanoflagellida
T0_74 T0h (17)
T4C3_86 T_T96h (17)
T4B3_41 C_T96h (17)
T4A4_33 UV+Nut_T96h (17)
clone OLI16108 AF107082
Chrysochromulina throndsenii AJ246277
T4B4_8 C+Nut_T96h (17)
T4C4_34 T+Nut_T96h (17)
T4C3_40 T_T96h (18)
T4D4_35 TUV+Nut_T96h (19)
T0_73 T0h (20)
Prymnesium patelliferum L34671
T4B4_35 C+Nut_T96h (19)
Prymnesium calathiferum U40923
Chrysochromulina kappa AJ246271
T4A4_30 UV+Nut_T96h (19)
Haptophyceae
T4B3_44 C_T96h (30)
T4B4_27 C+Nut_T96h (30)
T4C4_23 T+Nut_T96h (30)
T0_78 T0h (30)
T0_79 T0h (32)
Cercozoa environmental sample AY821945
uncultured Cryothecomonas AY628370
Cercomonas ATCC50316 U42448
Heteromita globosa U42447
Lotharella vacuolata AF054890
Pedinomonas minutissima AF054832
Lotharella sp. AF076170
T4B4_9 C+Nut_T96h (33)
T4D3_5 TUV_T96h (33)
Cercozoa
T4A3_14 UV_T96h (34)
T4D4_17 TUV+Nut_T96h (34)
T4A4_15 UV+Nut_T96h (34)
T4B4_38 C+Nut_T96h (34)
Chaunacanthid sp. AF018158
eukaryote clone OLI11015 AJ402332
Uncultured marine acantharean DH147-EKD17 AF290072
Acantharea
T4B4_25 C+Nut_T96h (35)
T4C4_18 T+Nut_T96h (35)
T4B3_35 C_T96h (35)
T4A3_18 UV_T96h (35)
T4A4_5 UV+Nut_T96h (35)
T4D4_21 TUV+Nut_T96h (35)
T4B3_5 C_T96h (36)
T4C3_69 T_T96h (36)
T4A4_32 UV+Nut_T96h (36)
T4B4_22 C+Nut_T96h (36)
Pirsonia diadema AJ561114
Pirsonia verrucosa AJ561113
Pirsonia
Eucampia antarctica X85389
T0_91 T0h (24)
Papiliocellulus elegans X85388
Skeletonema costatum X85395
Aulacoseira distans X85403
T4B4_39 C+Nut_T96h (22)
T4D4_42 TUV+Nut_T96h (23)
T4C3_19 T_T96h (21)
T4D3_30 TUV_T96h (21)
Rhizosolenia setigera M87329
T4A3_43 UV_T96h (25)
T4B4_16 C+Nut_T96h (25)
Bacillariophyta
T4B4_23 C_T96h (37)
T4D3_7 TUV_T96h (37)
T4C3_9 T_T96h (37)
T4C4_9 T+Nut_T96h (37)
T4A4_10 UV+Nut_T96h (37)
T4B3_34 C_T96h (37)
T4D4_20 TUV+Nut_T96h (37)
T4A3_27 UV_T96h (37)
T4B4_37 C+Nut_T96h (38)
Hyphochytrides
T0_55 T0h (39)
Cafeteria roenbergensis L27633
T4D4_30 TUV+Nut_T96h (42)
Bicosoecida
uncultured marine eukaryote DQ337351
Labyrinthuloides haliotidis U21338
T4A3_42 UV_T96h (40)
Thraustochytrium striatum AB022112
Labyrinthuloides minuta L27634
T4A4_19 UV+Nut_T96h (41)
Labyrinthulida
0.10
